# Decellularized pericardium tissues at increasing glucose, galactose and ribose concentrations and at different time points studied using scanning X-ray microscopy

**DOI:** 10.1107/S2052252521005054

**Published:** 2021-06-03

**Authors:** Cinzia Giannini, Liberato De Caro, Alberta Terzi, Luca Fusaro, Davide Altamura, Ana Diaz, Rocco Lassandro, Francesca Boccafoschi, Oliver Bunk

**Affiliations:** a Institute of Crystallography, National Research Council, Bari, 70126, Italy; b Department of Health Sciences, University of Piemonte Orientale, Novara, Italy; c Tissuegraft srl., Novara, Italy; d Paul Scherrer Institut, Villigen PSI, 5232, Switzerland

**Keywords:** microdiffraction, decellularized tissue models, SAXS, WAXS, X-ray scanning microscopy, diabetes, collagen, glycation

## Abstract

Diseases like widespread diabetes or rare galactosemia may lead to high sugar concentrations in the human body, thereby promoting the formation of glycoconjugates. Glycation of collagen, *i.e.* the formation of glucose bridges, is nonenzymatic and therefore cannot be prevented in any other way than keeping the sugar level low. It relates to secondary diseases, abundantly occurring in aging populations and diabetics. However, little is known about the effects of glycation of collagen on the molecular level. We studied *in vitro* the effect of glycation, with d-glucose and d-galactose as well as d-ribose, on the structure of type 1 collagen by preparing decellularized matrices of bovine pericardia soaked in different sugar solutions, at increasing concentrations (0, 2.5, 5, 10, 20 and 40 mg ml^−1^), and incubated at 37°C for 3, 14, 30 and 90 days. The tissue samples were analyzed with small- and wide-angle X-ray scattering in scanning mode. We found that glucose and galactose produce similar changes in collagen, *i.e.* they mainly affect the lateral packing between macromolecules. However, ribose is much faster in glycation, provoking a larger effect on the lateral packing, but also seems to cause qualitatively different effects on the collagen structure.

## Abstract 2

Decellularized pericardium tissue exposed to solutions of sugars like glucose, galactose and ribose could potentially be a model system for diseases like diabetes and the effects of aging. Scanning X-ray diffraction and scattering reveals the different nano-structural changes of collagen induced by glucose, galactose and ribose.

## 1. Introduction   

Diabetes mellitus is the most prevalent chronic disorder in the world. In 2019, the World Health Organization highlighted that almost 1.5 million deaths are due to diabetes and that it will become the seventh cause of death in ten years. Data extracted from the 2017 Global Burden of Disease Study gives a projection of 1.59 deaths in 2025 (Lin *et al.*, 2020 ▸). It is *de facto* a pandemic disease [consider that COVID-19 has had almost 1.8 million deaths, as reported by Huang *et al.* (2021 ▸) in January 2021] that not only heavily burdens the national health system of each nation but is also estimated to lead to a reduction in workforce and productivity of over 80 million lost person-years due to the early deaths (Lee & Veres, 2019 ▸). The escalating rate of diabetes has been driven by the genetic susceptibility of certain ethnic groups, human-environment changes, sedentary lifestyles, rapid socio-economic development and nutrition transition. In particular, in developing countries more than 80% of deaths are caused by diabetes mellitus, associated cardiovascular diseases, cancer and respiratory disease (Walker *et al.*, 2018 ▸; Mathers & Loncar, 2006 ▸), and there is a negative effect on fertility.

The classic clinical manifestation of diabetes is hyperglycemia, *i.e.* a high glucose concentration in the blood due to the altered insulin sensitivity, which exposes tissues to long-term high concentrations of sugar, consequent macro/micro-vascular pathologies and multi-organ damage. Thus, plasma-glucose (PG) criteria are commonly used to diagnose diabetes. Three criteria are crucial, with just two of these being required to be fulfilled to diagnosis diabetes for patients without classic symptoms. One of these PG criteria is the fasting PG (FPG), where fasting is settled as the absence of caloric intake for 8 h. The FPG has a critical point value of ≥126 mg dl^−1^ (*i.e.* 1.26 mg ml^−1^), corresponding to ≥7.0 mmol l^−1^. The 2 h PG value after a 75 g oral anhydrous glucose tolerance test is often associated with the FPG, and when its value is ≥200 mg dl^−1^, *i.e.* 2 mg ml^−1^, corresponding to 11.1 mmol l^−1^, it is a marker of prediabetes or diabetes (American Diabetes Association, 2020 ▸).

Diabetes is characterized by heterogeneous progression and clinical symptoms and is classified as follows. Type 1 diabetes is also called ‘juvenile diabetes’ as it is a common chronic pathology in childhood, although it can be identified at different ages. The disease is characterized by the autoimmune destruction of pancreatic insulin-secreting β-cells islets and life-long exogenous insulin-replacement dependence. At the initial stage, the disease is asymptomatic, but with the presence of autoantibodies (Knip *et al.*, 2010 ▸). The progression leads to the impaired glucose tolerance giving rise to clinical symptoms only in the last stage including the classic hallmarks: polyuria, polydipsia, polyphagia, metabolic imbalance and overt hyperglycemia (Insel *et al.*, 2015 ▸).

Type 2 diabetes is a multifactorial pathology with a slow and late onset. Over 450 million people are affected by type 2 diabetes, and, according to the International Diabetes Federation, it has been estimated that the number will rise to 700 million by 2045. There is a strong correlation between the incidence of type 2 diabetes and the western diet. It is a high caloric diet characterized by a great amount of saturated fats and simple carbohydrates that contributes to an increase of blood glucose, circulating free fatty acids, triglycerides and very low density lipoproteins that increase the inflammatory state of the organs through the release of proinflammatory molecules, *i.e.* interleukin 6, interleukin 1 and tumor necrosis factor alpha (Chatterjee *et al.*, 2017 ▸; Schwartz *et al.*, 2016 ▸). In this type of diabetes the main feature is hyperglycemia, due to the inability of insulin to maintain glucose homeostasis.

Diabetes is a heterogeneous family of pathologies. The members include not only the most common (type 1 and type 2) but others that are less common but equally severe. Early detection has also played a crucial role in the prognosis of the patients, apparently it can be as important as the glycemic control. Indeed, in subjects with controlled glycemic level, clinical trials show the presence of a ‘metabolic memory’ of the pre-existing hyperglycemia in the body that induces anomalies and the progression of diabetes complications (Holman *et al.*, 2008 ▸).

Thus, the metabolic memory is sustained by the changes induced by the hyperglycemia within the body, such as inflammation, reactive oxygen species (ROS) and the formation of non-enzymatic advanced glycation end-products (AGEs) by the non-reversible crosslink reaction between small sugars, in particular glucose, fructose and ribose, and lipids and proteins. In particular, glycation causes proteins to have an increased deposition and structural modification that stiffens the tissue and disrupts the normal cell interactions, leading to chronic inflammation and tissue damage.

As glycation is a non-enzymatic reaction, it cannot be controlled, and the formation of AGEs is proportional to the blood-sugar concentration. Starting with the AGEs accumulation inside tissues, hyperglycemia-induced ROS promote the formation of receptors for AGEs ligands (Yao & Brownlee, 2010 ▸). AGEs have a pivotal role in the morpho-functional deficiency of connective tissues, establishing intra- and inter-molecular non-elastic crosslinks with proteins and altering molecular and cellular functions. In 2019, Lee and Veres observed the reduction of tendon stretch after the yield point when tissue was exposed to a high ribose concentration and hypothesized that a high number of crosslinks avoid the intermolecular sliding, which is the basis of plasticity (Lee & Veres, 2019 ▸).

An important target of glycation is type 1 collagen. It is one of the main targets of glycation in the body due to its long half life (one to two years in bone, ten years in skin) and ubiquitous localization within connective tissues, making up 25–35% of all body proteins (Zimmet, 1999 ▸; Sell & Monnier, 2012 ▸).

In this framework, small- and wide-angle X-ray scattering (SAXS/WAXS) scanning microscopies have proved to be the ideal techniques to inspect the collagen triple helix, to analyze changes in its hierarchically organized architecture, to detect pathologic markers in diseased tissues, and even for the investigation of collagen-based medical devices (Terzi *et al.*, 2020 ▸). These label-free techniques have been employed by the authors in the past to inspect the morphological and structural alteration of collagen type 1 tissues at above-molecular (SAXS) and sub-molecular (WAXS) level in keratoconus (Sibillano *et al.*, 2016 ▸), diabetes mellitus (Giannini, Terzi *et al.*, 2019 ▸), coxarthrosis (Giannini, Siliqi *et al.*, 2014 ▸), aneurysms (Giannini, Ladisa *et al.*, 2019 ▸) and breast cancer (Vanna *et al.*, 2020 ▸) affected tissues. Raw data have been transformed into quantitative microscopies and further into quantitative characteristic structural parameters by means of advanced methods, which combine statistical and crystallographic approaches for high-throughput data screening and analysis. The synergic use of statistical and crystallographic methods allows one to quantitatively evaluate the tissue electron density, at above-molecular (SAXS) and sub-molecular (WAXS) level; to (co)localize soft tissues such as type 1 collagen, myofilament, elastin (Giannini, Ladisa *et al.*, 2019 ▸); determine collagen fiber direction, periodicity and electron density; differentiate normal from cross-linked collagen, the latter being denser and more aligned (Giannini, Siliqi *et al.*, 2014 ▸); identify the crystallographic origin of any possible additional expected or unexpected inorganic crystalline structure present in the tissues (Vanna *et al.*, 2020 ▸); and find the spatial relationship between inorganic phases with respect to the soft tissue components (Giannini, Ladisa *et al.*, 2019 ▸; Vanna *et al.*, 2020 ▸). Here, the same approach will be used in an *in vitro* study to explore the effect of d-glucose, d-galactose and d-ribose sugars on decellularized bovine pericardium type-1 collagen tissues kept in solution at increasing concentrations (0, 2.5, 5, 10, 20 and 40 mg ml^−1^), and incubated at 37°C for 3, 14, 30 and 90 days. One of the advantages of decellularized pericardium tissue for such studies is that it is a rather pure collagen matrix without, for example, muscle tissue.

Without pretending to give a comprehensive collection of the experiments realized so far on type 1 collagen by using synchrotron radiation techniques, it is worth noting the relevant works by Bertinetti *et al.* (2015 ▸) on collagen-based mineralized tissues, Meek & Knupp (2015 ▸) on corneal structure, Al-Jawad *et al.* (2007 ▸) on dental tissue, Karunaratne *et al.* (2016 ▸) on bone collagen and osteoporosis, Zimmermann *et al.* (2011 ▸) on bone aging, and Fessel *et al.* (2014 ▸) on AGEs in tendons.

## 2. Experimental   

### 2.1. Tissue preparation   

Decellularized bovine pericardia were obtained at increasing monosaccharide concentrations according to the following procedure. Bovine pericardia were obtained as byproducts of cattle slaughter in food industry, kindly provided by Gavazza 1913 Spa (Asti, Italy), and then decellularized, as described elsewhere (Giannini, Ladisa *et al.*, 2019 ▸; Giannini, Terzi *et al.*, 2019 ▸), following a chemical and enzymatical protocol in three steps: (*a*) 1 *M* NaCl, 8 m*M* 3-[(3-cholamido­propyl)­dimethyl­ammonio]-1-propane­sulfonate (CHAPS) detergent and 25 m*M* ethyl­enedi­amine­tetra­acetic acid (EDTA); (*b*) 1 *M* NaCl, 1.8 m*M* sodium do­decyl sulfate (SDS) and 25 m*M* EDTA; and (*c*) 6.4 µ*M* de­oxy­ribonuclease I from bovine pancreas (Sigma–Aldrich), 0.1 *M* MgCl and 0.9 *M* NaCl. After the decellularization process, samples were sterilized in 0.2% peracetic acid for 2 h. *In vitro* glycation was obtained as follows: decellularized matrices were soaked in different glucose, galactose and ribose solutions, at increasing concentrations [(1) = 0 mg ml^−1^, (2) = 2.5 mg ml^−1^, (3) = 5 mg ml^−1^, (4) = 10 mg ml^−1^, (5) = 20 mg ml^−1^, (6) = 40 mg ml^−1^]. Glucose concentrations beyond 10 m*M* (18 mg ml^−1^) are known to mimic the diabetic environment (Layton, 2015 ▸; Ito *et al.*, 2017 ▸). The samples were incubated at 37°C for 3, 14, 30 and 90 days. Carbohydrate solutions were renewed every seven days to avoid samples drying. At each time point, the final glucose and galactose concentrations in the matrices was evaluated using a Glucose Assay Kit (Abnova, Germany) and a Galactose Assay Kit (Sigma–Aldrich, Italy). Briefly, a sample of 5 mm was cut from each glucose- and galactose-treated matrix, subsequently lyophilized for 16 h, weighed, and hydrolyzed in 500 µl of a 75 m*M* NaCl, 25 m*M* EDTA pH = 8, 1% SDS and 100 µg ml^−1^ of proteinase K solution for 16 h. Then, 50 µl of the glucose-treated hydrolyzed matrix was added to an equal volume of Abnova Assay Reaction Mix and incubated for ten minutes, protected from light. Similarly, 50 µl of galactose-treated hydrolyzed matrix was added to an equal volume of Master Reaction Mix in a 96-well plate and incubated for 30 min, protected from light. At the end, both sample types were analyzed through fluorescence spectroscopy (Victor X4 Multilabel Plate Reader, Perkin Elmer, Italy) with an excitation wavelength of 540 nm and an emission wavelength of 590 nm. In order to measure the adsorbed monosaccharides quantity, concentrations were normalized with the weight of their respective analyzed matrices: the monosaccharide quantity obtained through spectroscopy was divided by the weight of tissue used for the kit, obtaining the monosaccharide concentration per mg of tissue.

### 2.2. SAXS, WAXS scanning microscopies   

SAXS and WAXS scanning microscopy data were collected at the cSAXS beamline of the Swiss Light Source in Villigen, Switzerland (Bunk *et al.*, 2009 ▸), with the same experimental setup described in a previous work (Giannini, Ladisa *et al.*, 2019 ▸; Giannini, Terzi *et al.*, 2019 ▸). The main components are a liquid N_2_ cooled fixed-exit Si(111) monochromator with a bendable second crystal for horizontal focusing, a dynamically bendable mirror for the rejection of higher X-ray energies and vertical focusing, a sample holder which allows it to accommodate up to 144 samples (see Fig. S1 of the supporting information) on a motorized 2D translation stage, a 7 m long evacuated flight tube for SAXS data collection, and a Pilatus 2M detector (Henrich *et al.*, 2009 ▸). For the WAXS measurements, the flight tube was removed and the detector was moved close to the sample position, see Table 1 ▸ for the detector distances used in SAXS/WAXS. The tissues were studied in ultralene sachets. Briefly, the sachets were sterilized by 30 min incubation in ethanol 70%(*v*/*v*) and three-times washed in phosphate-buffered saline (PBS) to remove the ethanol. Each tissue was cut into 1 × 1 cm pieces and placed in a sachet, with a drop of sterile PBS with 0.05% penicillin/streptomycin solution, and then closed. The whole procedure of sample preparation was performed under sterile conditions. The data collection was performed in continuous vertical lines, *i.e.* the sample moves at a constant speed of step size over exposure time vertically while the detector records data frames. The details of the experimental setup are summarized in Table 1 ▸. SAXS and WAXS 2D data were calibrated by silver behenate (SAXS) and NIST SRM640b (WAXS), and folded into 1D profiles, after integration in 16 azimuthal segments. For each sample, 4131 SAXS and 4131 WAXS 2D data frames were recorded. Examples are shown in Fig. S2.

## 3. Data analysis   

### 3.1. Relevant length scales   

Among the 28 proteins known as collagens, type 1 collagen is a fibrous protein hierarchically organized at sub-molecular scales from polypeptide to fibrils (Orgel *et al.*, 2006 ▸), accessed by WAXS, and at above-molecular scales from fibrils to microfibrils, accessed by SAXS (Terzi *et al.*, 2020 ▸). The models mostly referred to in literature are 7/2 (Okuyama *et al.*, 2006 ▸; Okuyama, 2008 ▸; Okuyama *et al.*, 2012 ▸) and 10/3 (Rich & Crick, 1961 ▸).

The collagen-forming polypeptide microfibrils contain triple-helix domains, ∼306 nm long and 1.5 nm wide, which fill the space in a staggered assembly resulting in an above-molecular structure which contains gap (0.54*D*) and overlap (0.46*D*) regions, and repeat with a periodicity *D* = 65–67 nm, along the fiber axis (Sherman *et al.*, 2015 ▸).

Therefore, the length scales of interest at the sub-molecular scale (WAXS) are: the meridional (along the fiber axis) length scale of 0.29 nm, corresponding to the distance between adjacent amino-acid residues, projected along the central axis of the helical structure, *i.e.* one third of the 0.86 nm unit height (Terzi *et al.*, 2020 ▸; Rich & Crick, 1961 ▸) and the equatorial (perpendicular to the fiber axis) length scale of 1.5 nm (Orgel *et al.*, 2001 ▸), corresponding to the distance between laterally spaced molecular triple helices. Furthermore, the length scales of interest at the above-molecular scale (SAXS) are: the meridional (along the fiber axis) length scale of *D* ≃ 65–67 nm (Sherman *et al.*, 2015 ▸), *i.e.* the periodicity of the staggered nanoscale assembly, which depends on the hydration state of collagen (Bertinetti *et al.*, 2015 ▸) and the equatorial (perpendicular to the fiber axis) length scale of 100–150 nm, *i.e.* the typical fibril diameter. We interpret the equatorial scattering peak in an inter-fibril way, *i.e.* we calculate the length scale as 2π/*q*. Interpreting this peak as part of the shape transform of cylindrical fibers (Goh *et al.*, 2005 ▸; Eikenberry *et al.*, 1982 ▸) yields smaller numerical values but does not affect the interpretation of relative changes – if any, as this particular peak is not relevant to the discussion below.

### 3.2. Segmentation   

To identify characteristic features for each sample and thereby outlier samples, the 1D azimuthally averaged WAXS and SAXS patterns were statistically analyzed by means of a signal-classification method (Lutz-Bueno *et al.*, 2018 ▸) to extract the least-correlated profiles. In the present context of homogenous samples, we used this method as quality control to check for changes of the collagen structure in parts of a sample, potentially related to issues like sample drying. As an example, in Fig. S3(*a*) four such profiles are displayed which are quite similar because the sample is laterally homogenous, derived from the SAXS data relevant to a control sample among the analyzed tissues. The entire dataset was clustered accordingly into four subsets displayed in the score plot as a function of the three principal components: PC1, PC2 and PC3 shown in Fig. S3, directly providing the relative abundance of each of the colored profiles in the whole dataset. Fig. S3 displays the distribution, pixel by pixel, of the representative signals within the explored area. This method allows one to (i) screen across a large dataset and extract a few relevant profiles to be deeply analyzed with crystallographic methods, and (ii) determine pixel by pixel, within the microscopy, the relative abundance of the selected profiles in the imaged sample area and their spatial distribution within the sample.

**Figure 1:**
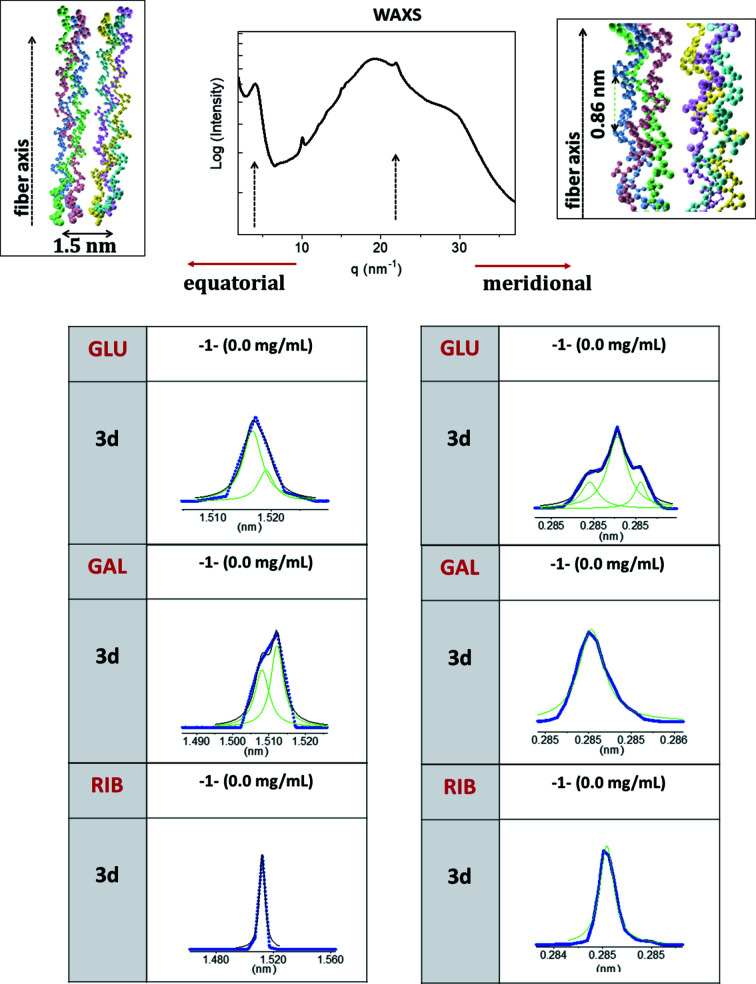
The above-molecular 1.5 and 0.86 nm distances are depicted in the molecular model of collagen, and the corresponding diffraction peaks at *q* = 2π/1.5 nm and *q* = 2π/(0.86 nm/3) for the third order of 0.86 nm are marked in a typical azimuthally averaged WAXS profile (top). For each of the 4131 WAXS frames recorded for each sample, the position of these peaks has been determined, providing the statistical population for extracting a few characteristic structural parameters for each sample. Furthermore, histograms of the above-molecular peak positions mapped across samples immersed in the specified sugar concentrations (zero, *i.e.* control samples in this example) for three days (3 d) are shown (bottom). The histograms of the WAXS equatorial peak, corresponding to the 1.5 nm distance, are depicted in the left column and the histograms of the meridional peak, corresponding to the 0.86 nm distance, are depicted in the right column.

Fig. 1 ▸ (top) shows a typical WAXS profile, extracted using segmentation. It contains the equatorial peak at *q* ≃ 4 nm^−1^, corresponding to the ∼1.57 nm length scale, and the meridional peak at *q* ≃ 22 nm^−1^, corresponding to the ∼0.286 nm length scale. Both peaks change across the entire microscopy as a function of the local glycation-affected collagen structure.

The segmentation analysis allows us to screen across identifying samples with SAXS or WAXS profiles that differ from the bulk set. This is shown in Fig. S4, where the SAXS profiles for samples incubated in the three sugars are compared with the profile measured on a control tissue without sugar (red line). This comparison indicates that most of the SAXS data are qualitatively similar to the control one, whereas striking differences, not only in the intensity but also in the periodicity of collagen peaks, occur for a few galactose profiles (see Fig. S5 for a plot of these three quite distinct galactose SAXS profiles).

### 3.3. Single-peak histogram analysis   

In the single-peak analysis, for each of the length scales identified in Section 3.1, the peak position, width and intensity of a Gaussian curve on *q* ^−*n*^ background was determined for each point across the entire raster-scanned area of 4 (vertical) × 2.5 (horizontal) mm. For this peak fitting, the SAXS and WAXS data integrated over the full azimuth was used. The 81 × 51 = 4131 peak positions for each sample are reported as histogram plots in Fig. S6 for WAXS and Fig. S7 for SAXS. There is one panel for each combination of the three sugars studied, *i.e.* glucose, galactose and ribose, the six sugar concentrations [(1) = 0 mg ml^−1^, (2) = 2.5 mg ml^−1^, (3) = 5 mg ml^−1^, (4) = 10 mg ml^−1^, (5) = 20 mg ml^−1^, (6) = 40 mg ml^−1^] and the four incubation times (3, 14, 30 and 90 days).

To illustrate the histogram analysis, we report in Fig. 1 ▸ the case of zero sugar concentration and three days of incubation time. Each histogram is fitted by one or more Gaussian functions, to determine for that sample with a certain sugar concentration and incubation time the characteristic length scale corresponding to a WAXS peak position. When more Gaussians were used, an average value was derived weighting the peak position, derived from each Gaussian, by the area under the peak. The full width at half-maximum (FWHM) of the histogram peak was determined as the average value of the FWHMs of the multiple Gaussian peaks weighted in the same way as carried out for the positions. This FWHM is used as a measure for the uncertainty in the length-scale evaluation.

### 3.4. Theoretical model to describe the SAXS meridional profile   

**Figure 2:**
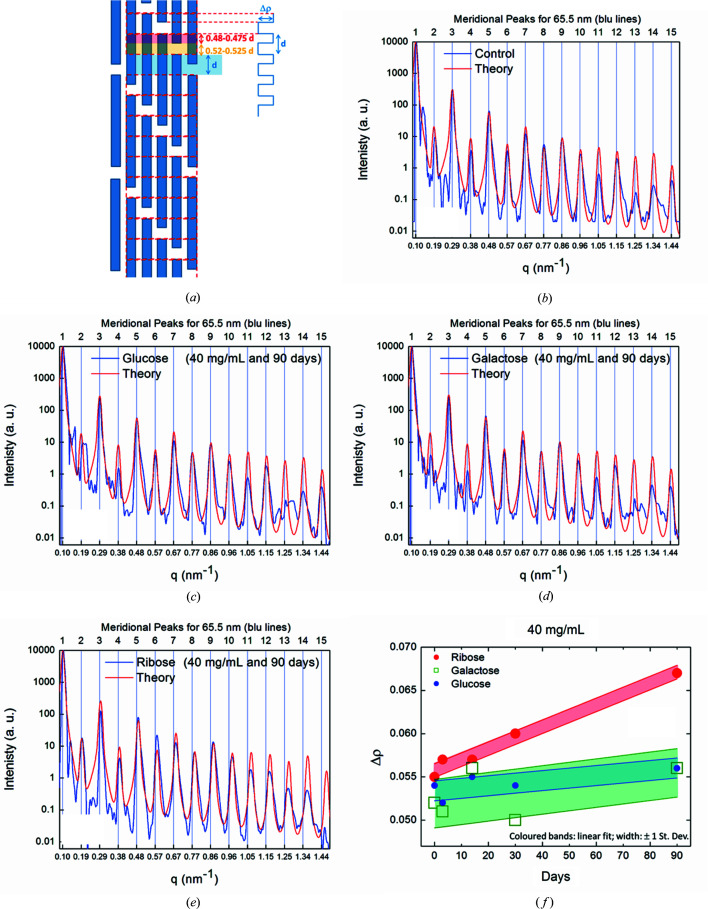
(*a*) A model of the microfibrillar arrangement and thereby electron-density distribution along the collagen fibers used to describe the SAXS data. These data are shown in experimental SAXS profiles (blue curves), extracted by averaging all profiles for (*b*) the control sample, (*c*) glucose, (*d*) galactose and (*e*) ribose (data for sugars refer to 40 mg ml^−1^ and 90 days), and compared with the theory (red curves). (*f*) Electron-density difference Δρ in the overlap region of length *d*, normalized with respect to the ρ_Ave_ value, as a function of sugar incubation time. The number of periods that interfere, determined by the comparison with the theory, is *N* = 10, for all the experimental data.

The staggered assembly of type 1 collagen, described in Section 3.1, leads to meridional SAXS profiles, see Fig. S3 for an example, which contain several Bragg peaks (00*l*) with integer *l*, related to the nanoscale periodicity *D* ≃ 63–67 nm along the fiber axis (Ottani *et al.*, 2002 ▸). One could determine an electron-density profile using a constrained phase-retrieval approach (Bunk *et al.*, 2007 ▸). However, constraints are required to reduce the number of free parameters. This implies a bias on the obtained retrieved solution. Conversely, we consider a model-based fitting procedure helpful for gaining an understanding of the fundamental structural effects like the average electron-density difference between the overlap and gap regions. As a first approximation, the axial electron density can be modeled with a 1D periodic step-like electron-density function (Madhurapantula & Orgel, 2017 ▸), see Fig. 2 ▸(*a*). For this, the axial period *D* is divided into two parts of constant electron density: ρ_1_, which extends for σ*D*, and the complementary electron density ρ_2_ with extension (1 − σ)*D*. Here, the excess electron density is defined as the offset with respect to the electron density ρ_0_ of the surrounding matrix. The number *N* of periods that interfere is limited both by the coherence of the impinging radiation and by the extension of the long-range order of the collagen structure. In the following, (ρ_1_, σ*D*) refers to the overlap region, while [ρ_2_, (1 − σ)*D*] to the gap region.

In a kinematic approximation, the scattered amplitude is given by the square modulus of the Fourier transform of the electron density (Guinier, 1994 ▸). The interference term due to the *N* periods is given as

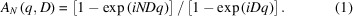

This term is modulated by a diffraction term, due to a single period *D*:

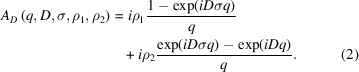

Here, 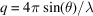 is the axial component of the scattering vector along the fiber axis.

The SAXS intensity along the meridional direction is proportional to

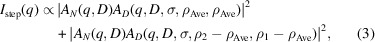

with 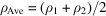 and ρ_2_ − ρ_Ave_ = −(ρ_1_ − ρ_Ave_) ≃ Δρ. The first term of equation (3) describes the monotonous intensity’s decrease, proportional to *q* ^2^. The second term describes the interference peaks, *i.e.* meridional Bragg peaks (see Fig. 5 of Suhonen *et al.*, 2005 ▸), when the condition 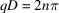 is satisfied, with *n* integer.

Multiple scattering and defects may lead to substantial damping of the interference peaks in the axial (meridional) direction. In fact, the contrast of the interference peaks is proportional to Δρ. However, the step-like electron density is an approximate description of the true electron density, an approximation which holds better at lower spatial resolution (lower *q* values). The true electron density changing less abruptly than the approximated step-like electron density will lead to a reduction of the interference peaks of higher order. To take all the above listed factors into account, a suitable damping function is introduced:

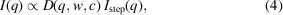

with a Lorentzian damping function *D*(*q*, *w*, *c*) whose minimum value at higher *q* is limited by a constant *c*, *i.e.*


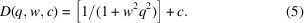

The step-like function, describing the electron-density discontinuities between the overlap and gap regions, is characterized by ideal sharp interfaces: in the range of only one pixel the electron density goes from the minimum to the maximum value. The damping function given by equation (5) takes into account the higher-frequency-cutoff effects of the finite width with which, in real structure, the electron density goes from the average value in the gap to the average value in the overlap region and *vice versa*. Indeed, our analysis aims to recover the average Δρ between overlap and gap regions and its dependence by glycation processes, neglecting its influence on the fine structure of the collagen electron density.

In Fig. 2 ▸, examples are shown for measured SAXS intensities – without the monotone contribution which corresponds to the ρ_Ave_ term of equation (3) and decreases as 1/*q* ^2^ – as the average over all data points measured for one sample in comparison with the intensities calculated using the model just described. In Figs. 2 ▸(*b*), 2 ▸(*c*), 2 ▸(*d*) and 2 ▸(*e*) the experimental data (blue curves) are plotted together with the best fits (red curves) for an untreated control and for the highest sugar concentration of 40 mg ml^−1^ [glucose in 2 ▸(*c*), galactose in 2 ▸(*d*) and ribose in 2 ▸(*e*)]. The electron-density differences Δρ determined by all the fits are plotted in 2 ▸(*f*).

A fit routine was written for this purpose. The fit parameters of the model are: *D*, *N*, σ, ρ_Ave_, Δρ, *w* and *c* [see equations (1)–(3) [Disp-formula fd3]]. First, all initial values have been attributed – namely, *D* is determined by the higher-order Bragg diffraction peaks’ position, *N* is estimated by the FWHM of the peaks, σ has a nominal value of 0.46 (Orgel *et al.*, 2001 ▸), ρ_Ave_ = 1, Δρ = 0 and *D*(*q*, *w*, *c*) = 1 (*w* = *c* = 0). Then, the routine finds the minimum of the least squares between experimental and theoretical data at each of the following steps. (i) Δρ is increased, in steps of 0.001. (ii) The damping function is added [multiplication factor in equation (4)], starting with *w* = *c* = 0 and varying *w* in steps of 0.1 and *c* in steps of 0.01. (iii) The fit proceeds by alternating a variation of Δρ – step (i) – and a variation in the damping function – step (ii) – until the minimum is found. (iv) *D* and σ values are refined, allowing variations of 0.1 nm in *D* and variations of 0.001 in σ. (v) Steps (i)–(iv) are repeated, refining the values of Δρ, *w*, *c*, *D* and σ that were previously obtained, until the error does not change anymore and convergence is reached. Usually, this requires few iterations (the maximum number of iterations of the whole fitting procedure is fixed to ten). Final values of *w* and *c* range in the interval 8–10 nm and 0.04–0.07, respectively, for all the fits shown in Fig. 2 ▸. Final *R* ^2^ values (1 − *R* ^2^ is defined as the ratio of the variance of the fit with respect to experimental data over the variance of the data with respect to the mean value), obtained for the fits shown in Fig. 2 ▸, excluding data before the first scattering peak, range between 0.74 and 0.8. Thus, the step-like model allows one to explain 0.74–0.80 of the observed experimental data variations as a function of *q*. The complementary value, 1 − *R* ^2^, equal to 0.2–0.26, is the unexplained variance of data, caused both by experimental measurement errors and by the approximate theoretical model (step-like electron density) used to fit data. Therefore, the approximation of a step-like electron-density function can explain ∼75–80% of the observed peak variability of WAXS data. In fact, the square root of *R* ^2^ ranges in the interval 0.87–0.91. Even if we assume that all the residual value of 1 − (*R* ^2^)^1/2^ is due to only one of the fitting parameters, *e.g.* Δρ, we still have a systematic error on its determination, due to the approximation of the theoretical model – *i.e.* the step-like function – with respect to the real electron density of the collagen structure, of the order of 10%, since 1 − (*R* ^2^)^1/2^ ranges from 0.09 to 0.13. Fig. 2 ▸(*f*) shows a variability in Δρ as large as 20%. In fact, in the residual value of 1 − (*R* ^2^)^1/2^ there are errors due to experimental measurement and caused by the determination of all the other fitting parameters. We can conclude that the systematic error due to the assumed approximate theoretical model, the step-like electron density function, is surely much smaller than 1 − (*R* ^2^)^1/2^, *i.e.* 10%. Therefore, within the experimental errors – the colored bands shown in Fig. 2 ▸(*f*) – the Δρ values derived by the step-like electron-density model can be considered a correct evaluation of the actual values of the real collagen structure under investigation.

**Figure 3:**
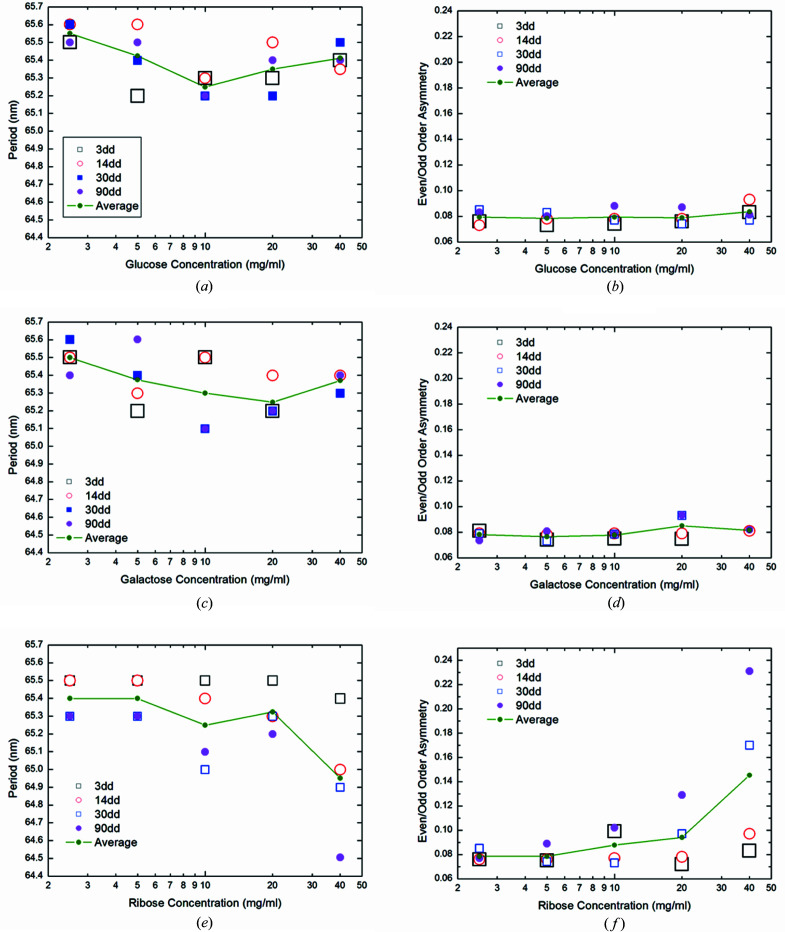
Periodicity along the axial direction determined in a model-dependent fit for glucose (*a*), galactose (*c*) and ribose (*e*), at increasing concentration and different incubation times. Asymmetry values of the nanoscale axial electron density for glucose (*b*), galactose (*d*) and ribose (*f*), at increasing concentration and different incubation times. The symbols correspond to fits of *I*(*q*) from the average across curves at these concentrations.

The period *D* has been determined both via the single-peak analysis described in Section 3.3 and via the model-dependent fit described in this section. The latter values are plotted in Figs. 3 ▸(*a*), 3 ▸(*c*) and 3 ▸(*e*).

### 3.5. Periodicity and asymmetry value along the micro-fibril axis   

The staggered arrangement of the triple-helix domains with a shift of ∼65 nm leads to a series of diffraction peaks in the meridional direction. Considering that the angular position of the peaks encodes the information on the micro-fibril periodicity, we reported in Fig. 3 ▸ the periodicity along the axial direction at increasing concentrations and different incubation times, as determined from the model-dependent fits for glucose [Fig. 3 ▸(*a*)], galactose [Fig. 3 ▸(*c*)] and ribose [Fig. 3 ▸(*e*)].

The relative intensity of the diffraction peaks varies with the electron density along the fiber axis. In case of hydrated and hardly glycated collagen, the even diffraction orders are strongly suppressed. To capture the deviation from this unperturbed state, we introduce an asymmetry value, calculated as the sum of the integrated peak intensities of all even diffraction orders starting at the fourth order divided by the sum of the peak intensities of all odd diffraction orders starting at the third order. The higher this asymmetry value, the stronger the deviation from the unperturbed state of a hydrated and non-glycated control sample. Asymmetry values are plotted for glucose [Fig. 3 ▸(*a*)], galactose [Fig. 3 ▸(*c*)] and ribose [Fig. 3 ▸(*e*)]. These values have been calculated based on the average SAXS profile for each sample.

## 4. Results   

### 4.1. Histogram analysis at the different length scales   

Figs. S9 and S10 display the four relevant length scales identified in Section 3.1 as a function of concentration and for different incubation times.

Perpendicular to the fiber axis, the ∼1.5 nm equatorial distance between molecular triple helices (Terzi *et al.*, 2020 ▸; Rich & Crick, 1961 ▸) is found to change in perhaps a similar way for glucose and galactose, with an initial dip, *i.e.* intermediate contraction, followed by a moderate increase. The absolute value stays below 1.6 nm for all combinations of sugar concentration and incubation time studied here. In contrast to this, a much stronger increase to 1.7 nm at the highest concentration and incubation time is observed for ribose.

Along the fiber axis, the meridional ∼0.286 nm amino-acids distance along the triple helix exhibits an initial peak, *i.e.* an intermediate swelling for glucose at low concentrations, followed by a moderate increase that in the case of longer incubation times may be interpreted as a second peak. Roughly similar behavior is observed for galactose and ribose. However, in the case of ribose, the increase in the first distance is not observed, *i.e.* the data as a function of ribose concentration resemble more a contraction followed by an intermediate swelling rather than two intermediate swellings.

The equatorial fibril diameter of ∼150 nm is spread across a wide range of values. There may be a tendency towards an initial dip, *i.e.* contraction with increasing sugar concentration, but the trends are quite heterogeneous.

The meridional ∼7.3 nm distance as the ninth order of the staggered repeat distance of *d* ≃ 65 nm exhibits an initial minimum as well. However, the trend towards higher sugar concentration and incubation time differs significantly in the case of ribose with a contraction to ∼7.16 nm compared with the glucose and galactose values of ∼7.28 nm. As this distance corresponds to the ninth order, the difference between ribose on the one hand and glucose and galactose on the other is ∼1 nm in the *d* spacing along the fiber axis, 64.4 nm versus 65.5 nm.

### 4.2. Sugar concentration integrated over time   

The visual inspection of trends of the equatorial and meridional distances in Figs. S9 and S10, as a function of sugar concentration and incubation time, let us assume that the effects of the sugar on the collagen structure depend on the sugar concentration and the duration over which the tissue is exposed to the sugar. This means that they depend on the sugar concentration integrated over time rather than the concentration alone. We therefore plotted the same trends also as a function of the sugar concentration integrated over time in Figs. S10 and S11, the latter for a zoom-in at small values. We cannot prove or disprove which variable best describes the changes in peak position but conclude that it may not be a simple linear relationship as assumed in these plots.

### 4.3. Axial electron density   

The asymmetry values plotted in Figs. 3 ▸(*b*), 3 ▸(*d*) and 3 ▸(*f*) exhibit a pronounced increase for the combination of high ribose concentrations and high incubation times, whereas the variation in this value is much smaller in the cases of glucose and galactose. This coincides with a shrinking of the *D* period for ribose at these conditions determined both via peak fitting, see Fig. 3 ▸(*e*), and independently via the statistical histogram analysis, see the bottom of Fig. S9. While the electron-density difference Δρ in the overlap region of length *d* increases as a function of sugar incubation time for all sugars, the effect is most pronounced in the case of ribose, see Fig. 2 ▸(*f*).

**Figure 4:**
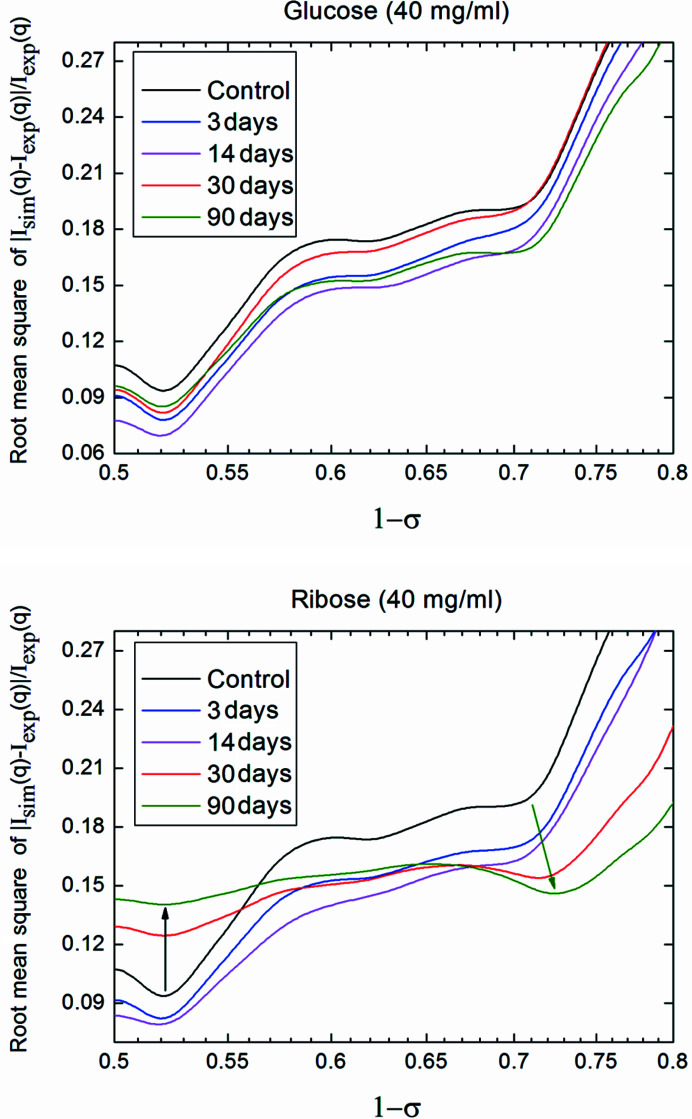
Variation of the mean square-root error between the theoretical curve and the experimental data as a function of the axial gap fraction (1 − σ), for ribose (top) and glucose (bottom), at the maximum concentration (40 mg ml^−1^) and different incubation times, leaving constant the other fitting parameters (*d* and *Δρ*).

Fig. 4 ▸ shows the variation of the mean square-root error between the calculated and measured SAXS intensities as a function of the gap/overlap fraction, for ribose and glucose, at the maximum concentration (40 mg ml^−1^) and different incubation times. The other fitting parameters *d* and Δρ are kept constant at the previously determined optimum values, assuming largely uncorrelated parameters. In Fig. 4 ▸, in the case of ribose, the minimum, which is found about an overlapping region (1 − σ) ≃ 0.52–0.525, becomes less pronounced with increasing incubation time (see the black arrow). Moreover, a second minimum region, at very large (1 − σ) values (see the green arrow), is more and more evident when the incubation time increases. Due to Babinet’s principle, we cannot be sure if larger (1 − σ) values correspond to longer overlap regions of the microfibrils and smaller gaps in between them, or *vice versa*. However, in view of the shrinking *D* period, larger overlap and smaller gaps are likely to be the case.

For glucose, the minimum around (1 − σ) ≃ 0.52–0.525 in Fig. 4 ▸ remains pronounced even at long incubation times in combination with the highest sugar concentration studied. The quality of the galactose sample is insufficient for such an analysis. Nevertheless, we observe for galactose samples that the values of asymmetry, see Fig. 3 ▸(*d*), the period, see Fig. 3 ▸(*c*), and the contrast density Δρ, see Fig. 2 ▸(*f*), exhibit less spread and variation than in the case of ribose. This means that the collagen structure is less effected by exposure to glucose and galactose than to ribose.

The anomalous profiles (the blue and green curves in Fig. S5) were singular cases, interpreted as the results of an imperfect preparation of that specific tissue or a dried sample, which were not further analyzed.

## 5. Discussion   

Thus, these *in vitro* experiments clearly indicate that glycation with glucose, galactose and ribose each affect type 1 collagen differently. The findings of the WAXS/SAXS experiments, described in Section 4, are summarized as follows.

(*a*) Expansion at the molecular scale perpendicular to the fiber axis (all the sugars). All the sugars affect the equatorial length scale of 1.5 nm, corresponding to the distance between laterally spaced molecular triple helices. Glucose and galactose exhibit an initial contraction of the lateral distance followed by an increase from 1.46 to 1.57 nm. In comparison, the increase rate is significantly higher for ribose, changing from 1.51 to 1.70 nm.

(*b*) Electron density along the fiber axis. The sugar that most affected the electron density along the fiber axis was ribose. The axial nanoscale periodicity reduces from *D* = 65.5 to *d* = 64.4 nm, confirming what was found (Gautieri *et al.*, 2017 ▸) in an *in vitro* ribosylation study of rat-tail collagen soaked in sugar for up to 12 incubation days. Conversely, the trends in the *d* nanoscale periodicity along the fiber axis are similar for glucose and galactose, with a slight decrease up to 10 mg ml^−1^ in the case of glucose and up to 20 mg ml^−1^ for galactose, followed by a recovery up to 40 mg ml^−1^. Ribose is also changing the axial electron-density distribution, as revealed by the odd/even peaks’ asymmetry values, indicating pronounced changes in the axial electron density for the highest sugar concentrations and longest incubation times. However, no pronounced change in the electron-density profiles along the meridional axis is found for glucose with respect to the control sample. The same is true for galactose, apart from a few profiles, which we attribute to a problem in the sample preparation. In addition, Δρ was also found to increase, due to ribose binding to collagen and thereby increasing the electron-density difference between overlap and gap regions. The linear fits shown in Fig. 2 ▸(*f*) indicate the time slope of the electron-density difference Δρ in the overlap region of length *d*, normalized with respect to the ρ_Ave_ value, as a function of sugar incubation time. For galactose and glucose, 90 incubation days causes an increment of ∼7% in the Δρ values with respect to those observed for untreated samples. For ribose, 90 incubation days causes an increment of ∼20% in the Δρ value with respect to that observed for untreated samples. Therefore, we estimate that the increment of the axial electron density due to glucose and galactose after 90 days is already measured after a one-third shorter period – about one month – with ribose. This temporal dilation is also evident by looking at the variation of the period and the even/odd asymmetry of the peaks, which are much lower for glucose and galactose.

d-Glucose (C_6_H_12_O_6_) and d-galactose (C_6_H_12_O_6_) have an identical molecular weight of 180.16 g mol^−1^ and a topological polar surface area of 110 Å^2^. This reflects in similar SAXS and WAXS results on the nanoscale structure. d-Ribose (C_5_H_10_O_5_) has a smaller molecular weight of 150.13 g mol^−1^ and a smaller topological polar surface area of 90.2 Å^2^. This relates to the faster increase in the equatorial (perpendicular to the fiber axis) distance between micro-fibrils of 1.5 nm with increasing ribose concentration, and in the more pronounced effect on the meridional electron-density profile in comparison with glucose and galactose. It is known that ribose has the ability to react with proteins to produce glycated derivatives, *i.e.* AGEs, more rapidly than glycation with glucose (Han *et al.*, 2011 ▸). In this respect, our data are the perfect experiment to prove this and we could conclude that ribose is a more dangerous sugar than glucose or galactose, at least with respect to the structural effect on type 1 collagen. In a recent work, abnormally high levels of d-ribose were found in the urine of patients with type 2 diabetes mellitus, together with d-glucose, suggesting that diabetic patients suffer from dysmetabolism of not only d-glucose but also d-ribose (Wu *et al.*, 2019 ▸).

## 6. Conclusions and perspectives   

The effect of glucose, galactose and ribose on the structure of collagen has been determined using scanning SAXS and WAXS microscopies. The parameter space has been sampled varying both sugar concentration up to 40 mg ml^−1^ and incubation time up to 90 days. The ∼1.5 nm lateral distance between molecular triple helices of collagen was found to increase from 1.46 to 1.57 nm for glucose and galactose, and from 1.51 to 1.70 nm for ribose. For ribose, a significant contraction of the nanoscale *D* period was also found, accompanied by an increase in the electron-density difference between overlap and gap regions and change in the gap-size fraction. This work may open the way to also using bovine pericardium, already used in the past (Sizeland *et al.*, 2014 ▸), as a model system to study the effects of glycation and ribosylation. It might have interesting perspectives considering that AGEs, due to their relevant toxicity, are known to promote host cell death and damage organs (Byun *et al.*, 2017 ▸) in several diseases (brain: Alzheimer, Parkinson, stroke; lung: idiopathic pulmonary fibrosis; heart: myocardial infarction; bone: osteoarthritis; liver: hepatic fibrosis, cirrhosis; kidney: diabetic nephropathy). Therefore, a large type of collagen-based pathologic tissues could be studied with the described approach to map, at different hierarchical levels, type 1 collagen accumulation and re-organization.

Finally, we expect this study is a starting point for well defined sugar-level/structure/property relationship studies relying on abundantly available secondary products of the food industries rather than ethically delicate and less well controlled studies based on human donors or animal models.

## Supplementary Material

Supporting information. DOI: 10.1107/S2052252521005054/ro5026sup1.pdf

## Figures and Tables

**Table 1:** Experimental details for the SAXS/WAXS experiment

Monochromatic X-ray beam	λ = 0.09124 nm, *E* = 13.589 keV
Beam size	25 µm (vertical) and 45 µm (horizontal)
Incident X-ray flux†	2.4 × 10^11^photons s^−1^
Sample to SAXS detector distance	7098 mm
Sample to WAXS detector distance	243.7 mm
Raster-scanned area on each sample	4 (vertical) × 2.5 (horizontal) mm
Step size, both horizontally and vertically	50 µm
Exposure time for one SAXS frame	0.4 s
Resulting no. of SAXS/WAXS data points for each sample	81 × 51 = 4131
Exposure time for one WAXS frame	0.3 s

† The incident flux was measured with a standard glassy carbon specimen (Allen *et al.*, 2017 ▸).
