# Supplementary material for: Decellularized pericardium tissues at increasing glucose, galactose and ribose concentrations and at different time points studied using scanning X-ray microscopy
Source: IUCrJ. 2021 Jun 3;8(Pt 4):621–32. doi: 10.1107/S2052252521005054 (PMC8256709; doi:10.1107/S2052252521005054)
Supplement: Supplementary file 1 [file m-08-00621-sup1.pdf]

IUCrJ

Volume 8 (2021)

Supporting information for article:

Decellularized pericardium tissues at increasing glucose, galactose and ribose concentrations and at different time points studied using scanning X-ray microscopy

Cinzia Giannini, Liberato De Caro, Alberta Terzi, Luca Fusaro, Davide Altamura, Ana Diaz, Rocco Lassandro, Francesca Boccafoschi and Oliver Bunk

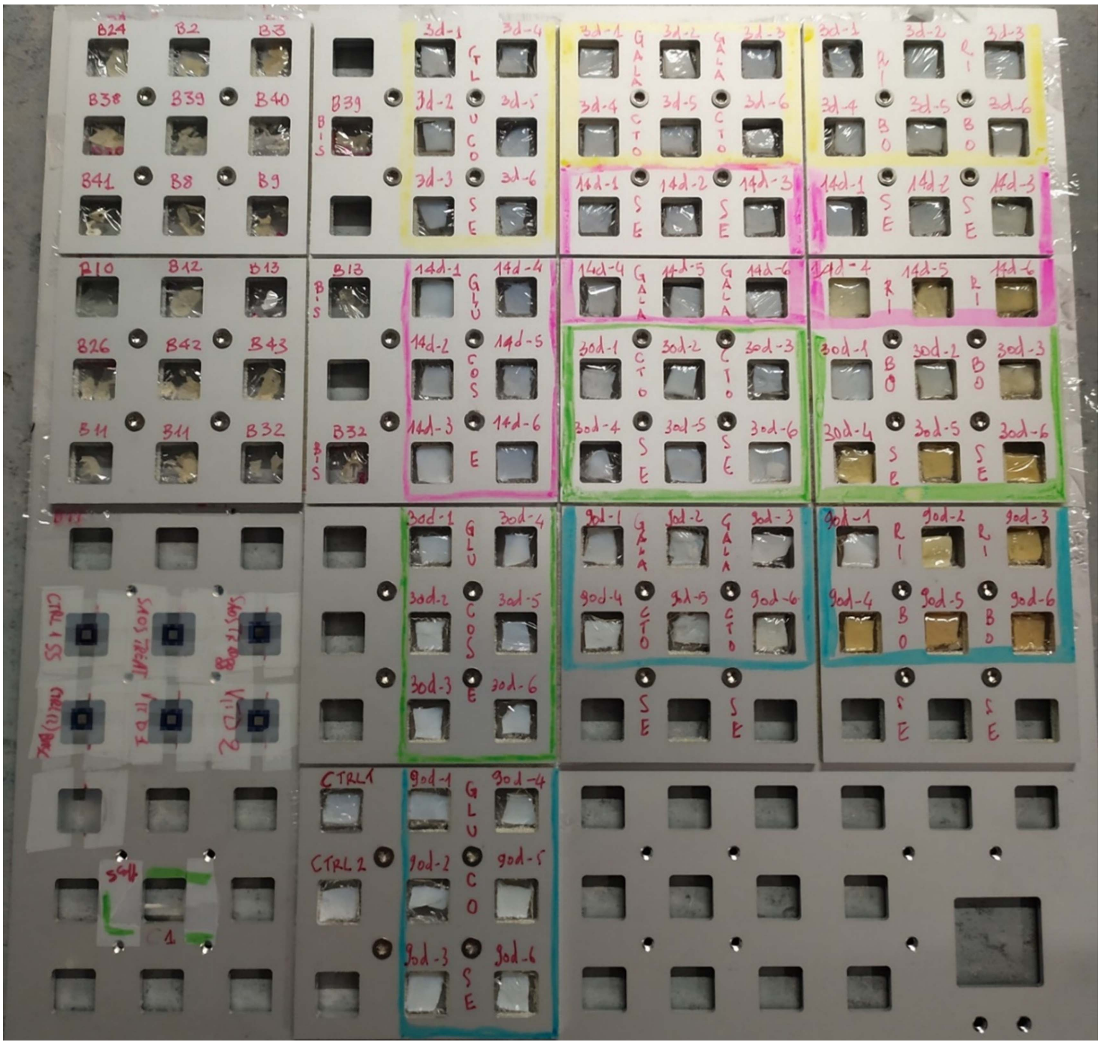

Figure S1 Picture of the sample holder for the SAXS/WAXS measurements. Each square aperture holds one sample to be raster-scanned through the X-ray beam.

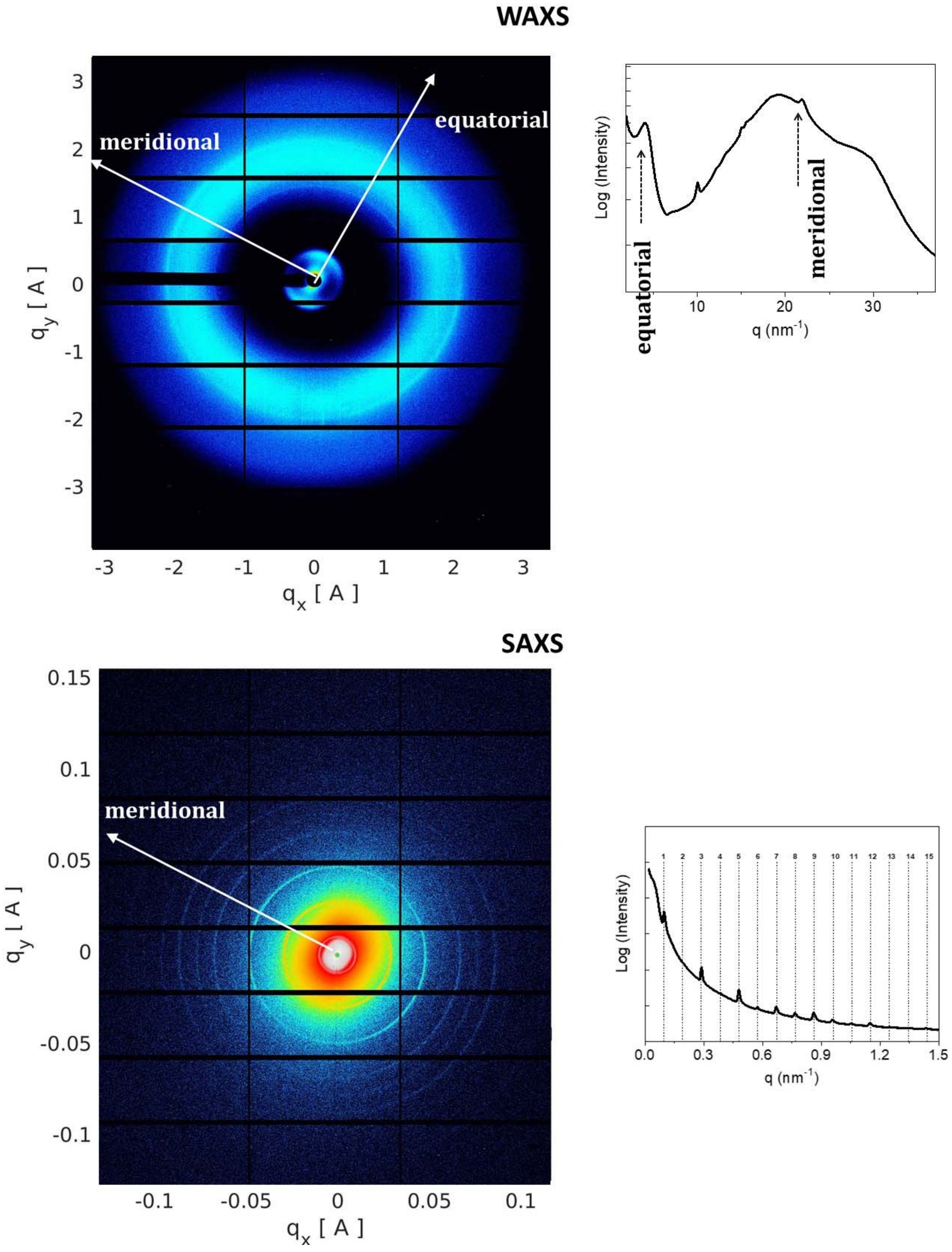

Figure S2 Examples for WAXS and SAXS data. On each sample, 4131 WAXS and 4131 SAXS data frames have been recorded. The 2D WAXS and SAXS data once centred and calibrated are folded in 1D WAXS and SAXS profiles for further analysis. The equatorial and meridional directions are here shown: the meridional is along the fiber axis and the equatorial is perpendicular to the fiber axis. The reader may refer to § 3.1. Relevant length scales for the meaning of each peak, or peak series.

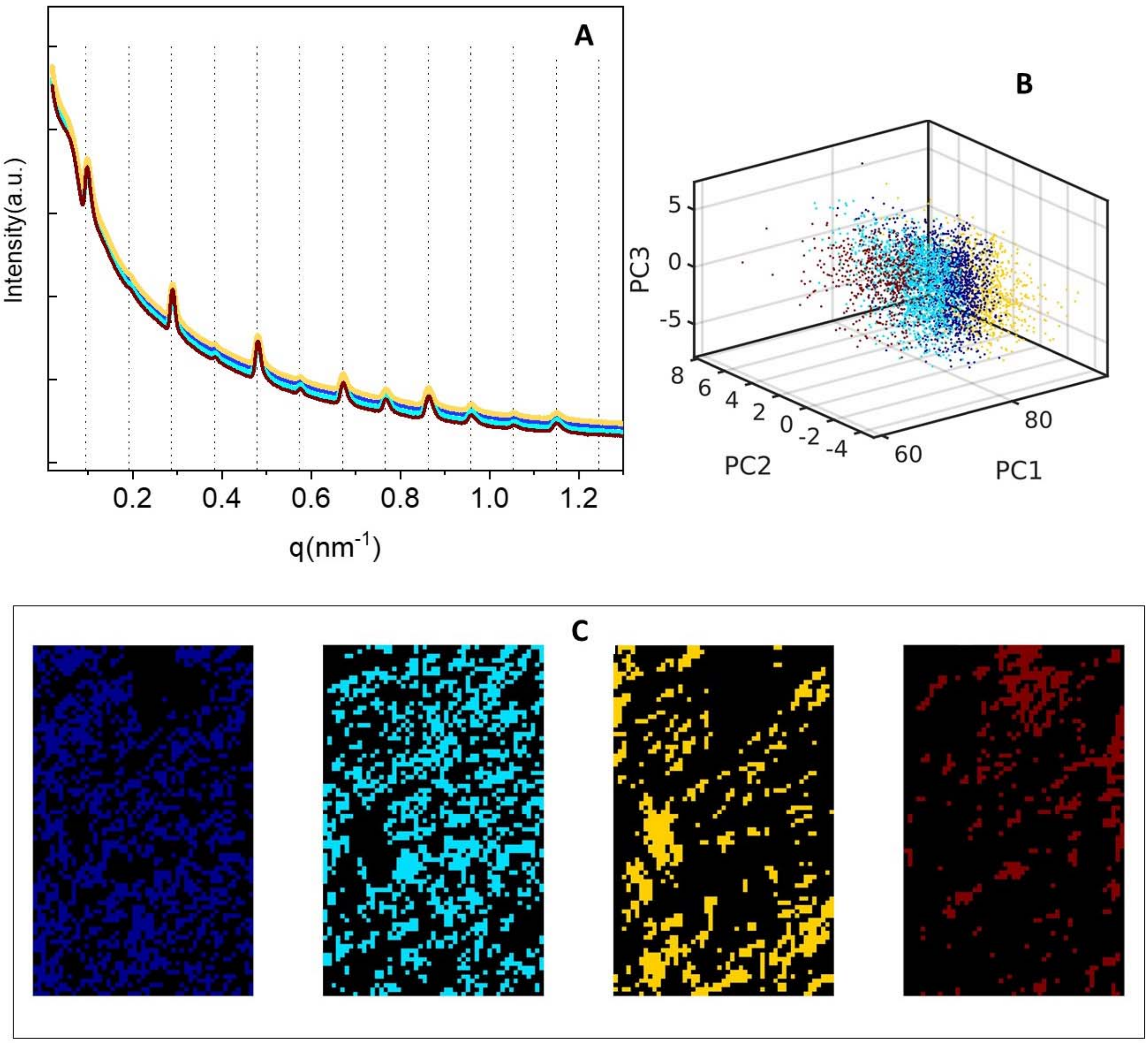

Figure S3 A) least-correlated SAXS patterns statistically selected by the signal-classification method in § 3.2. In the present case of a control sample, the four components are very similar, indicating the good homogeneity of the sample; B) clustering of the entire dataset into four subsets displayed in the score plot as a function of the three principal components: PC1, PC2 and PC3; C) distribution of the selected SAXS patterns in the explored area, allowing to trace back the spatial origin of each pattern.

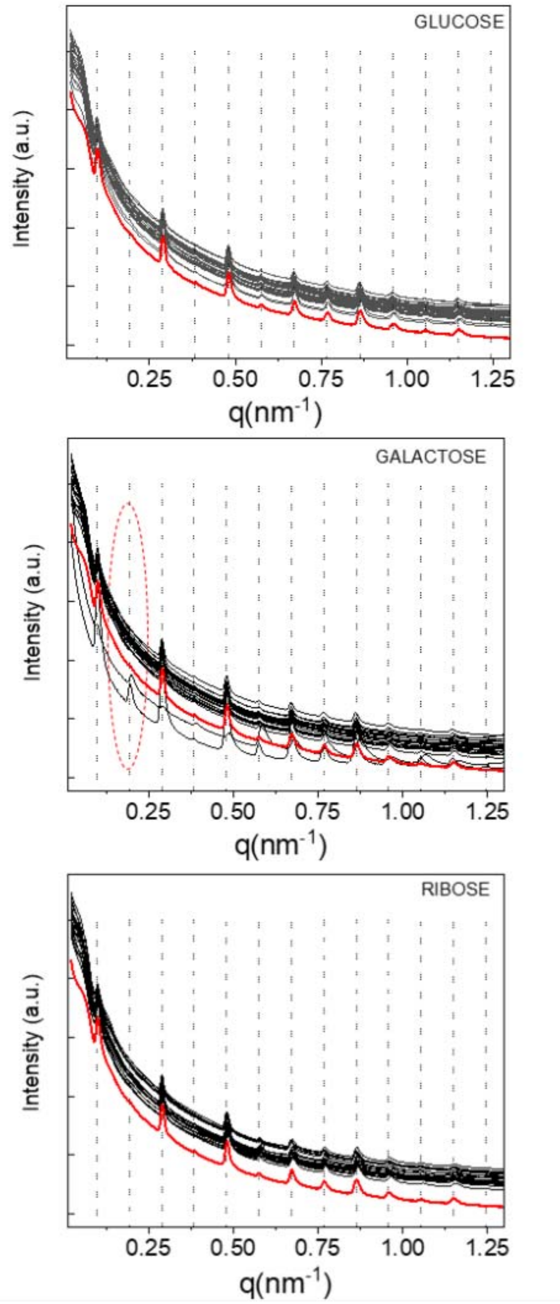

Figure S4 Examples for identifying characteristic profiles using segmentation. SAXS profiles, sorted by segmentation among glucose, galactose and ribose patterns and compared with the SAXS profiles measured on a control sample (red curve). The second peak intensity, which changes especially for a few galactose tissues, is highlighted by the red oval. Such outliers have been excluded from some of the analysis steps (see text).

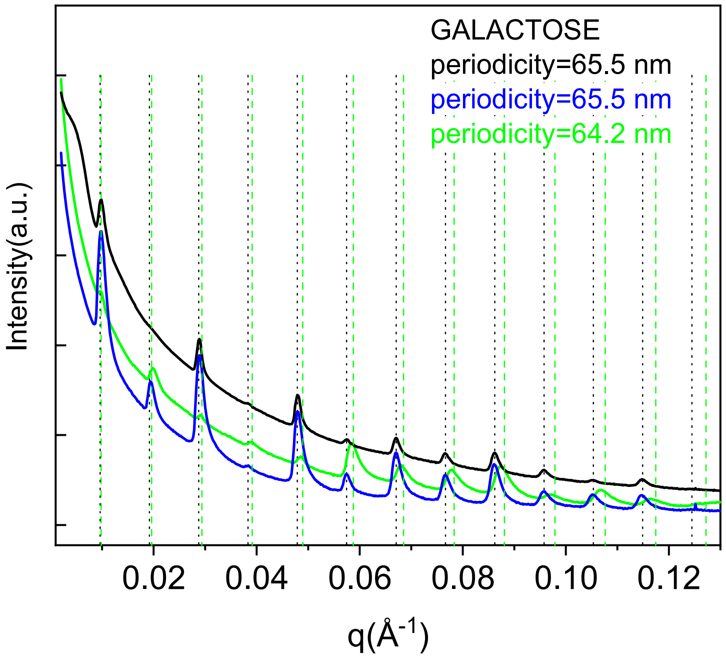

Figure S5 Examples for identifying characteristic profiles using segmentation and its use for quality control. Three galactose SAXS profiles, differing for the peak intensity (compare blue and black) as well as peak intensity and periodicity (compare green and black). The blue and green outliers may be attributed to sample drying or sample preparation issues, occurring for very few samples only that have been excluded from further analysis based on the results of this quality check.

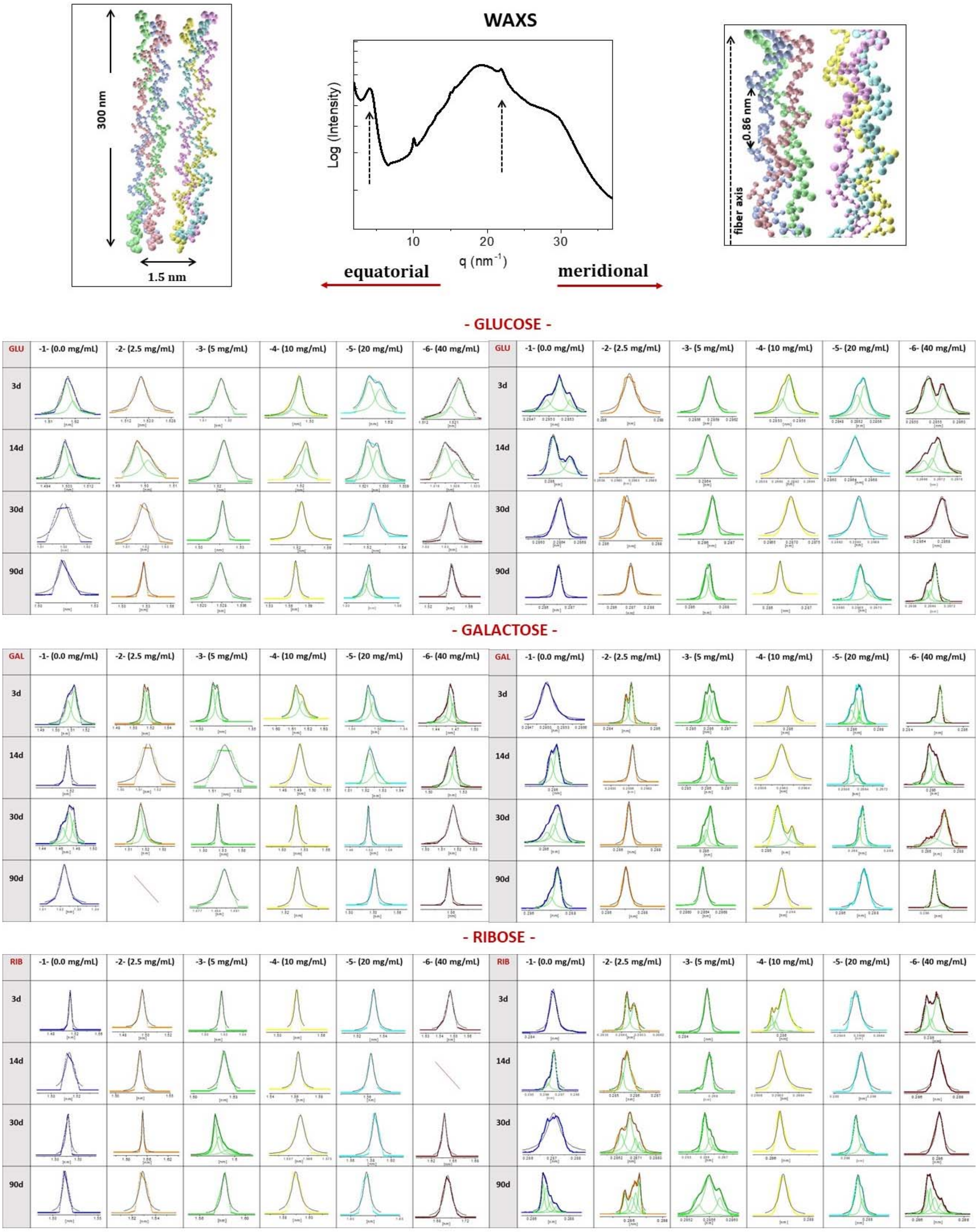

Figure S6 (top) Typical WAXS profile, extracted using segmentation (§3.2), which contains equatorial peak at q~4 nm^{-1} (~1.5 nm diameter of the triple-helix domain) and meridional peak at q~22 nm^{-1} (~0.286 nm length scale, 1/3 of the 0.86 nm axial rise between amino acids. (bottom) Histograms of equatorial (left) and meridional (right) WAXS peak positions as a function of concentration and incubation time for glucose, galactose and ribose. For each of the 4131 WAXS frames recorded per sample, the position of these peaks has been determined, providing the statistical populations (histograms) further analyzed.

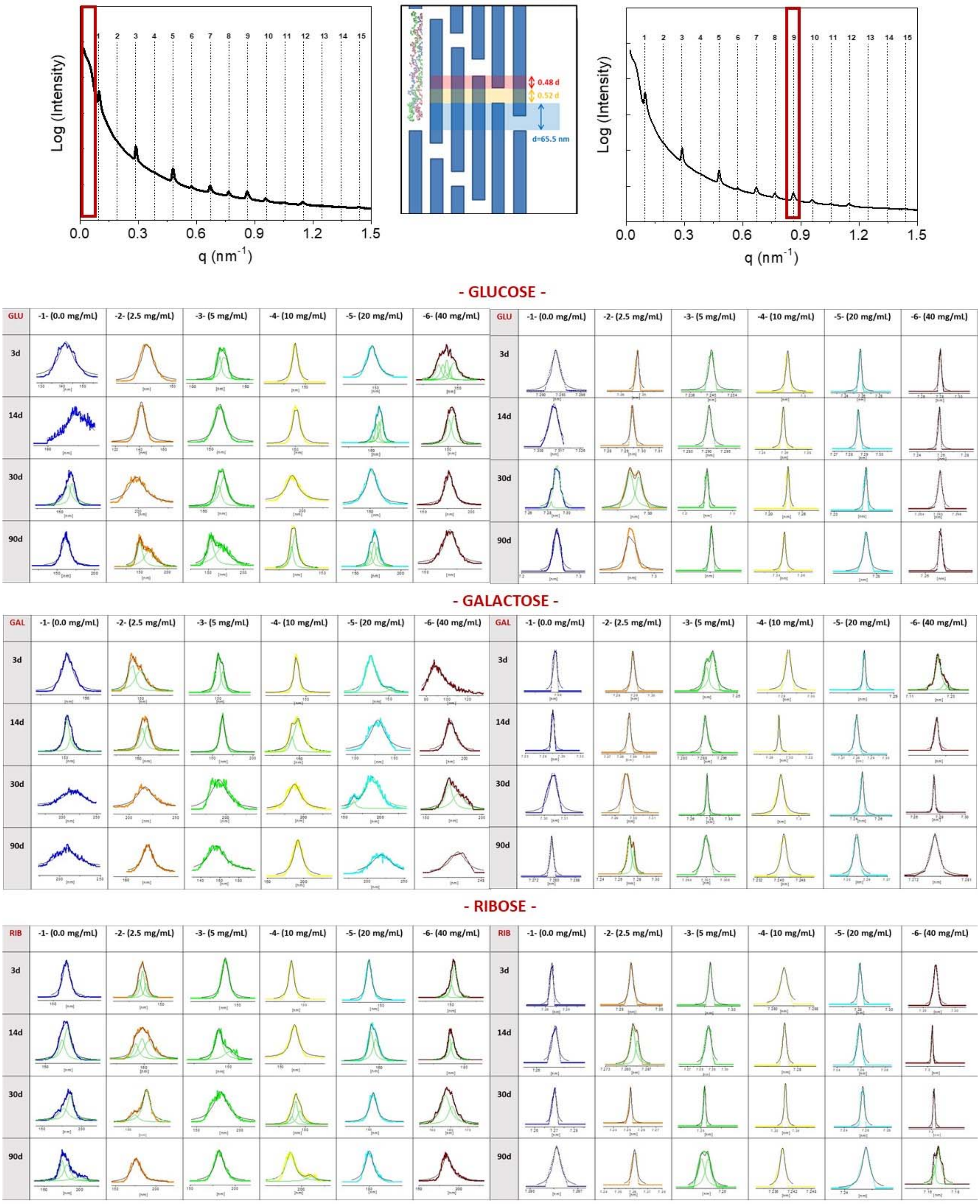

Figure S7 (top) Typical SAXS profile, extracted using segmentation (§3.2), which contains the equatorial peak at q~0.05 nm^{-1} (~125 nm length scale), and the meridional peak at q~0.864 nm^{-1} (~7.3 nm length scale, i.e. 9th order of the axial nanoscale periodicity of D~65.5 nm). (bottom) Histograms of equatorial (left) and meridional (right) SAXS peak positions as a function of concentration and incubation time for glucose, galactose and ribose. For each of the 4131 SAXS frames recorded for each sample, the position of these peaks has been determined, providing the statistical populations (histograms) further analyzed.

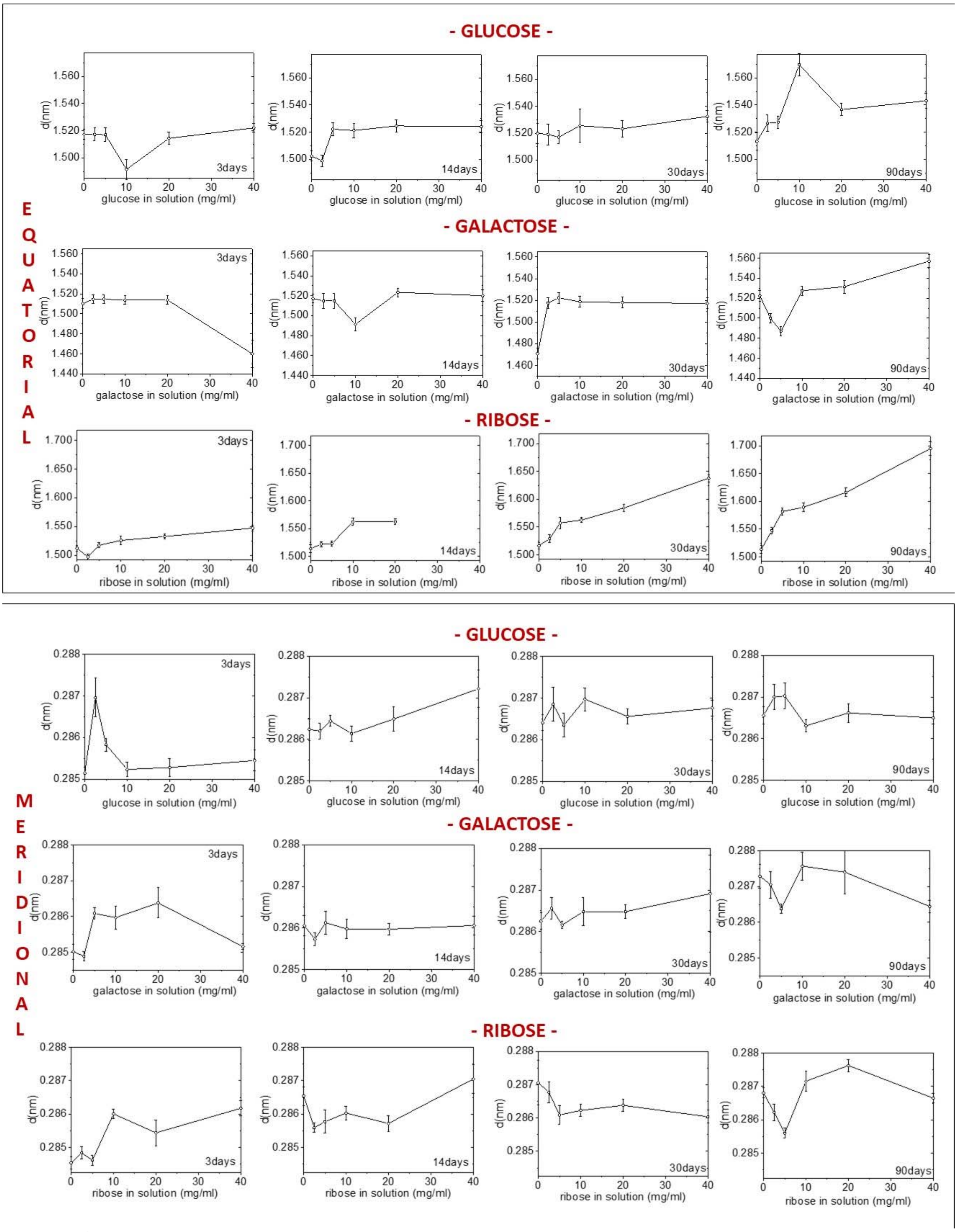

Figure S8 Length scales corresponding to WAXS peak positions determined from the histograms for each sample, i.e. combination of sugar type, concentration and incubation time. (top) The equatorial ~1.5 nm corresponding to the dimension of the triple-helix domain. (bottom) The meridional 0.286 nm corresponding to the third order of the rise between amino acids.

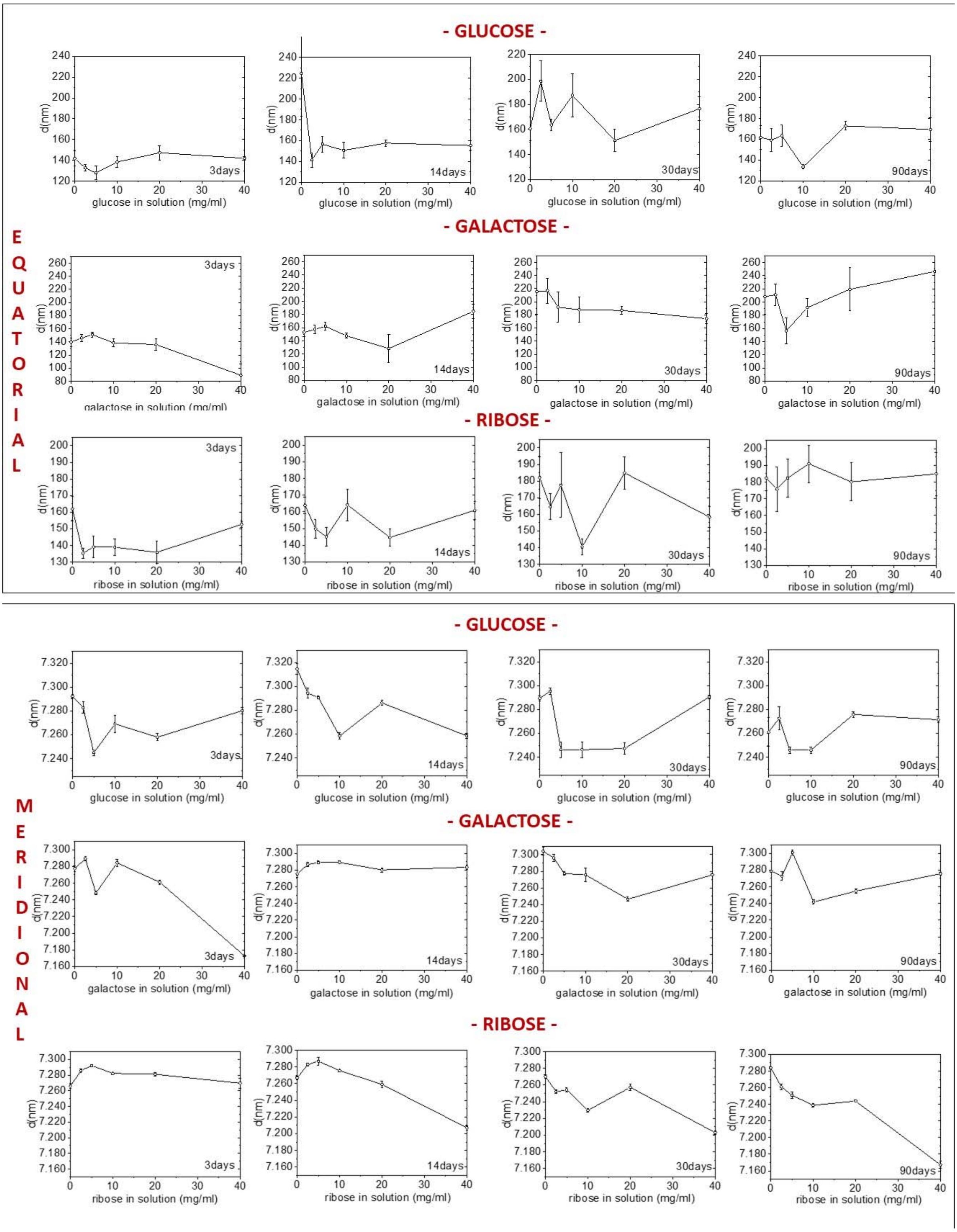

Figure S9 Length scales corresponding to SAXS peak positions determined from the histograms for each sample, i.e. combination of sugar type, concentration and incubation time. (top) The equatorial ~100-150 nm corresponding to the dimension of the micro-fibrils. (bottom) The meridional 7.3 nm corresponding to the ninth order of d~65-67 nm, the periodicity of the staggered nanoscale assembly.

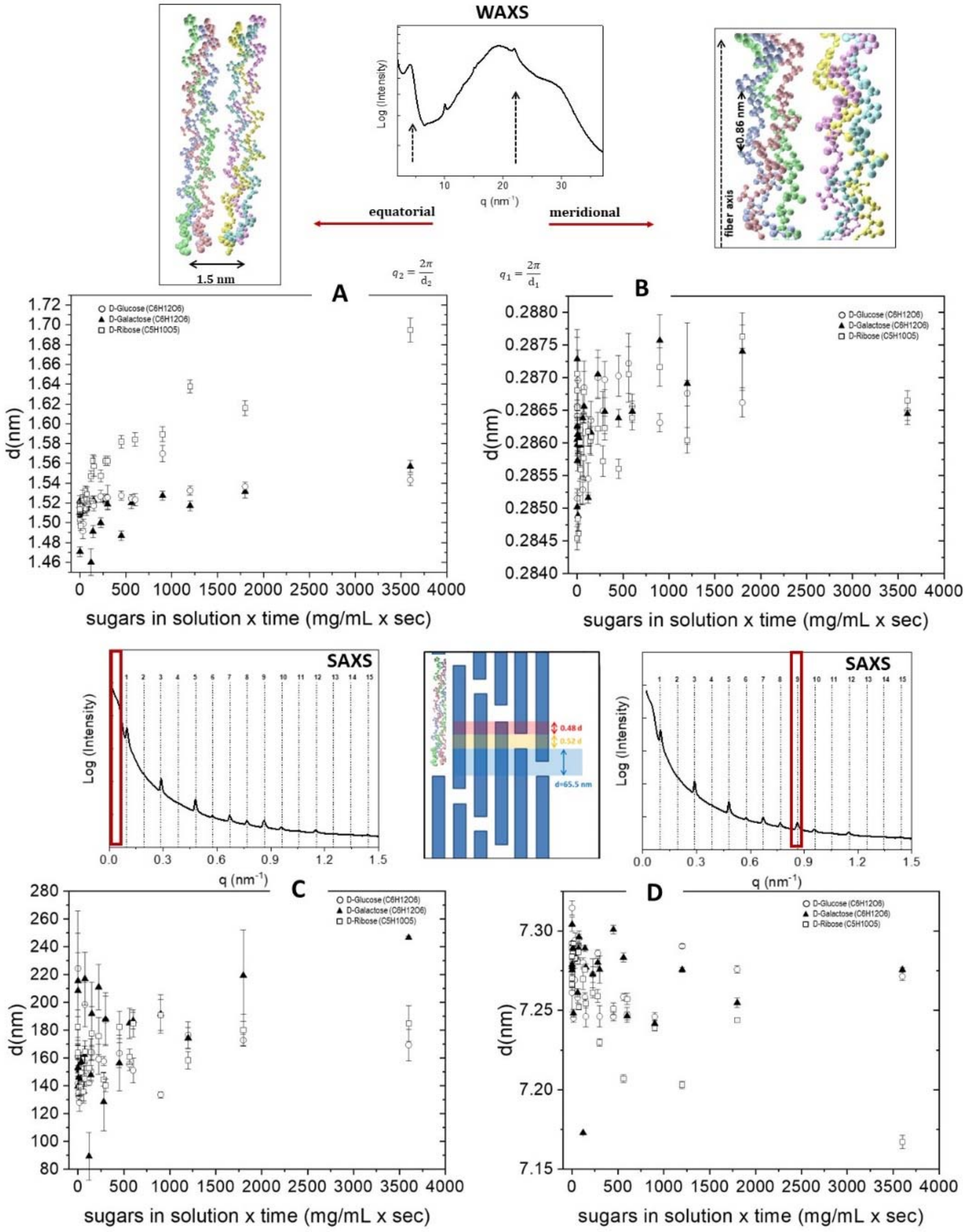

Figure S10 Equatorial WAXS (A) and SAXS (C) and meridional WAXS (B) and SAXS (D) peak-position and thus supramolecular distance as a function of the integrated sugar dose for glucose (open dots), galactose (full triangles) and ribose (open squares).

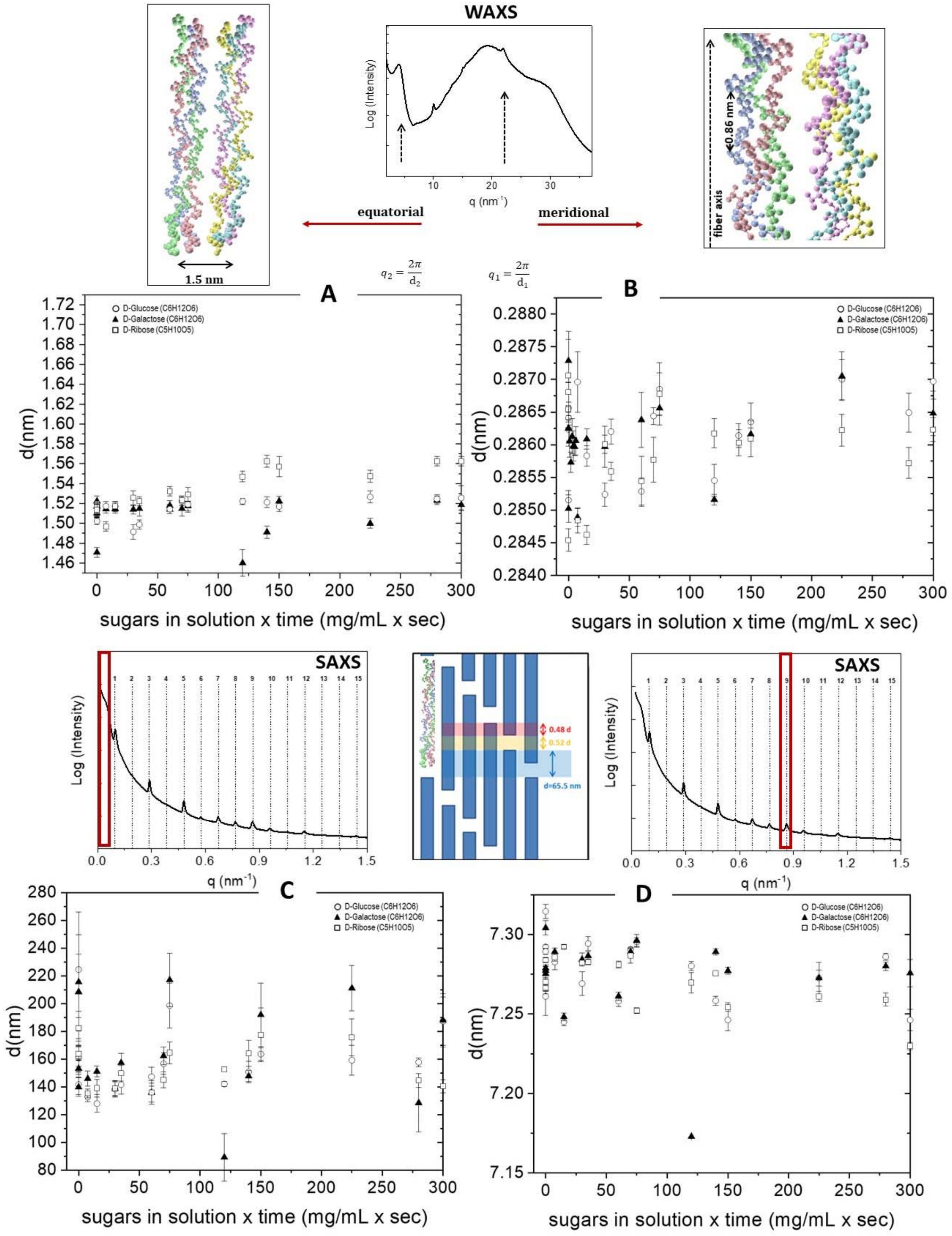

Figure S11 Initial trends of the equatorial WAXS (A) and SAXS (C) and of the meridional WAXS (B) and SAXS (D) peaks for the glucose (open dots), galactose (full triangles) and ribose (open squares).
